# A prognostic model correlated with fatty acid metabolism in Ewing’s sarcoma based on bioinformatics analysis

**DOI:** 10.1515/med-2025-1238

**Published:** 2025-08-07

**Authors:** Xianwei Chen, Yuqi Yang, Dongqi Li, En Ye, Bingjian He, Mingshu Yu, Jiankai Luo, Jing Zhang

**Affiliations:** Department of Orthopaedics, Peking University Cancer Hospital Yunnan Hospital, Yunnan Cancer Hospital, The Third Affiliated Hospital of Kunming Medical University, Kunming, Yunnan, 650118, P.R. China; Department of Pathology, Peking University Cancer Hospital Yunnan Hospital, Yunnan Cancer Hospital, The Third Affiliated Hospital of Kunming Medical University, Kunming, Yunnan, 650118, China; Department of Orthopaedics, Peking University Cancer Hospital Yunnan Hospital, Yunnan Cancer Hospital, The Third Affiliated Hospital of Kunming Medical University, Kunzhou Road 519#, Kunming, Yunnan, 650118, P.R. China

**Keywords:** Ewing’s sarcoma, fatty acid metabolism, bioinformatics, biomarker, prognosis model, immune infiltration

## Abstract

**Background:**

Ewing’s sarcoma (EWS) is a highly aggressive malignant tumor that originates from bone or soft tissue. To date, there is no established prognostic model for EWS tumor. This study aims to identify prognostic genes and develop a predictive model associated with fatty acid metabolism in EWS using bioinformatics analysis.

**Results:**

We analyzed the GSE17679 dataset and identified 25 differentially expressed genes related to fatty acid metabolism in EWS. A risk model composed of ACADM, ADH5, ACSL1, ELOVL4, ECI1, PPT1, and ACOT7 gene signatures was constructed. The AUC values at 3 and 5 years were both ≥0.7, indicating good predictive accuracy. GSVA analysis revealed significant differences in fatty acid metabolism pathway enrichment between high- and low-risk groups. Differential genes were primarily enriched in pathways such as fatty acid oxidation, lipid oxidation, lipid modification, and fatty acid degradation. Immune infiltration analysis showed significant differences in memory B cells, activated NK cells, and neutrophils between the two groups. Additionally, significant differences were observed in the expression of immune checkpoints such as HAVCR2 and LDHB. Immunohistochemistry and survival analysis further demonstrated that the expression of PPT1 and ACOT7 proteins was associated with the progression-free survival of EWS patients.

**Conclusions:**

We successfully constructed a prognostic model for EWS related to fatty acid metabolism genes. PPT1 and ACOT7 may serve as promising predictors for EWS prognosis.

## 1 Introduction

Ewing’s sarcoma (EWS) is the second most common primary bone tumor in children and adolescents, often originating in soft tissue. It is an extremely aggressive cancer, with a survival rate of 70–80% for patients with localized disease, but less than 30% for those with metastatic disease [1]. Over the past two decades, EWS survival rates have remained largely stagnant worldwide due to the lack of effective therapies [2,3]. In fact, most EWS patients already have micrometastases at the time of diagnosis. According to the WHO classification of bone tumors (2020), EWS is categorized as a small round cell sarcoma, primarily characterized by different EWS gene (EWSR1) fusions on chromosome 22q12 [4]. Although numerous biomarkers have been identified in EWS [5], the metastatic mechanisms of EWS remain unclear. Therefore, establishing a systematic prognostic model to predict EWS metastases is crucial for guiding early diagnosis and treatment.

While EWSR1-FLI1 is the driver gene of EWS oncogenesis, its expression often fails to predict EWS metastases due to influences from epigenetics, metabolomics, and other factors [6]. Cancer cells can often regulate their metabolism through multiple pathways. Compared to normal cells, cancer cells seem to be more likely to utilize energy through glycolysis and abnormal fatty acid metabolism due to the imbalance of the tricarboxylic acid cycle [1]. Recently, studies have demonstrated significant alterations in fatty acid metabolism in tumor cells [2,7]. Cancer cells rely on fatty acids as essential materials for cell synthesis, including cell membrane production, energy storage, and the synthesis of signaling molecules [3,8]. Fatty acid metabolism has been shown to be associated with prognosis in patients with rhabdomyosarcoma, breast cancer, lung cancer, and colon cancer [9,10,11,12]. A recent study also indicated that increased lipid peroxidation could lead to changes in S-phase progression and apoptosis in EWS cells [13]. These findings suggest that fatty acid metabolism may play a vital role in EWS tumor metastasis.

Therefore, leveraging public databases and bioinformatics approaches, this study comprehensively analyzed the prognostic value and potential mechanisms of fatty acid metabolism genes in EWS. The findings were further validated using immunohistochemical assays. This study provides a novel tool for predicting the prognosis of EWS patients and lays the groundwork for future research into the molecular mechanisms of fatty acid metabolism in EWS.

## 2 Research methods

### 2.1 Data construction, evaluation, and validation of the fatty acid metabolism-related gene (FAMG) risk model for EWS

We use EWS GSE17679 from the GEO database as a training set and the ICGC (BOCA-FR) as a testing set. A total of 92 FAMGs were obtained from the literature. Eighty-three FAMGs were detected in the GSE17679 dataset. Then, differentially expressed FAMGs (DE-FAMGs) were analyzed by the Limma R package (version 4.2) [4]. We set |log2FC| > 1 and *P* < 0.05. The FAMGs significantly associated with overall survival in EWS were identified using univariate Cox regression analysis (*P* < 0.05). These genes were included in the subsequent LASSO analysis. Next, we constructed the risk model by risk score (from multivariate Cox analysis). Patients were classified into two groups based on the median risk score. Kaplan–Meier survival analysis was used to compare survival outcomes between these groups. The predictive accuracy of the risk score model for overall survival in EWS patients was evaluated using time-dependent ROC curves.

### 2.2 Analysis of differential genes between high- and low-risk groups, GO and KEGG enrichment, and protein–protein interaction (PPI) network

High and low groups differentially expressed genes (DEGs) by “Limma” [4]. |log_2_FC| > 1 and *P* < 0.05 were set, and enrichment analysis was performed (clusterProfiler) [5]. The STRING V11.5 website was employed for PPI analysis (https://cn.string-db.org).

### 2.3 Analysis of immune infiltration and immune checkpoints in high- and low-risk groups

The EWSTIMATE algorithm was applied to infer infiltrating stromal cells and immune cells [6]. CIBERSORT [7] was used to predict differences in the levels of infiltrating immune cells. GSVA was used to quantify the enrichment scores of KEGG pathways and immune risk genes in various immune-related gene sets [8]. Subsequently, we analyzed the expression of immune checkpoints.

### 2.4 Immunohistochemistry

A total of 27 paraffin-embedded EWS tissue specimens were collected between May 2009 and June 2022 for immunohistochemical staining (Approval Number: KYLX2023-074). All patients were histologically diagnosed at Yunnan Cancer Hospital (Table 1). The follow-up time for patients ranged from 3 to 165 months (median: 22 months). We reviewed the relevant literature of seven genes (PPT1, ACADM, ADH5, ACOT7, ACSL1, ELOVL4, and ECI1) and selected five genes (PPT1, ACADM, ACOT7, ACSL1, and ECI1) that can obtain antibodies (Table S1) with high clinical application values. The tissue was fixed overnight, dehydrated, and embedded, and an immunohistochemical section was performed on 4 μm-thick sections. Using the SP immunohistochemistry two-step method, the sections were dewaxed, hydrated, subjected to antigen repair, and incubated. The sections were blocked with endogenous peroxidase and then washed. All antibodies were diluted according to the instructions. Digital pathological sections were scanned, and two experienced pathologists, blinded to the sample origins and patient outcomes, independently assessed each section. At least five high visual fields were observed, and the final immunoreactivity score (the intensity score * the extent score of stained cells) was calculated according to the German semiquantitative scoring system [9].

**Table 1:** General clinical data of 27 EWS patients

Clinicopathological characteristics	ACOT7		*P*	PPT1		*P*
	≥6	<6		≥6	<6	
**Sex**						
Male	8	5		7	6	
Female	7	7	0.42	9	5	0.44
**Age**						
<18	7	3		5	5	
≥18	8	9	0.23	11	6	0.36
**Enneking stage**						
II	10	8		12	6	
III	5	4	0.66	4	5	0.24
**Location**						
Limbs	8	7		11	4	
Trunk	3	2		2	3	
Pelvis	4	3	1.00	3	4	0.32


**Ethics and Consent:** We obtained approval from the Ethics Committee of the Third Affiliated Hospital of Kunming Medical University (KYLX2023-074) on 2023-04-21.We have applied for exemption from signing the informed consent form when approved by the ethics committee.

## 3 Results

### 3.1 Construction, evaluation, and validation of the EWS FAMG risk model

**Figure 1:**
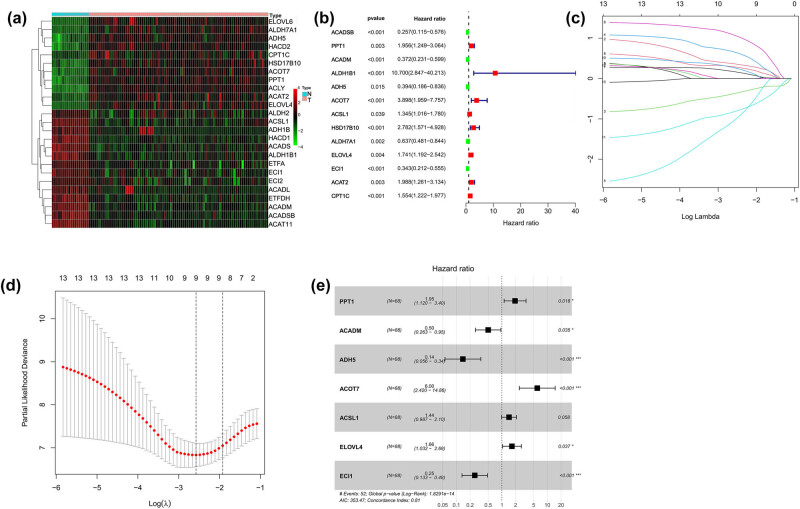
Construction of an FAMG risk model of EWS. (a) Heatmap of 25 differentially expressed fatty acid metabolism genes in tumors and normal tissues. (b) Thirteen genes were screened by univariate Cox regression analysis from FAMGs. (c) and (d) Nine genes were selected by LASSO analysis. (e) A prognostic model for seven genes was constructed by multivariate Cox regression analysis from FAMGs.

**Figure 2:**
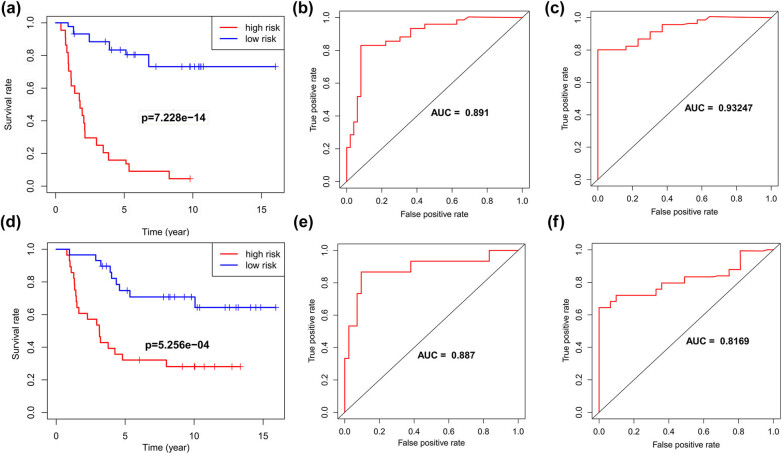
Survival analysis and ROC curves for training and testing sets. (a) Survival curves of the high- and low-risk score groups of the training sets. (b) and (c) ROC curves at 3 and 5 years for the risk model of training sets. (d) Survival curves of the high- and low-risk score groups of the testing sets. (e) and (f) ROC curves at 3 and 5 years for the risk model of the testing sets.

Using univariate Cox regression, LASSO regression, and multivariate Cox regression, a prognostic model based on seven markers was constructed. A total of 25 DE-FAMGs were identified, comprising 11 upregulated and 14 downregulated genes (Figure 1a, Table S2). In total, 13 genes associated with prognosis were screened (Figure 1b) and included in the LASSO analysis. In the LASSO analysis, 13 genes were included in the penalty. As the penalty coefficient lambda was varied, an increasing number of variable coefficients were compressed to zero. The lambda.min = 0.076 obtained the best performance, and lambda.Lse = 0.146 was selected as a simpler model without significantly reducing the model performance (Figure 1c and d). Finally, nine genes were selected and included in the multivariate Cox analysis. As shown in Figure 1e, seven genes related to prognosis (*P* < 0.05) were PPT1, ACADM, ADH5, ACOT7, ACSL1, ELOVL4, and ECI1. The results showed that prognosis was significantly better in the low-risk score group than in the high one in the training (*P* < 0.001, Figure 2a) and testing sets (*P* < 0.001, Figure 2d). We evaluated the model using the ROC curves. The results showed that both the AUC values at 3 and 5 years were ≥0.7, indicating a decent precision accuracy (Figure 2b, c, e and f).

### 3.2 Enrichment analysis on DEGs between the high- and low-risk groups

**Figure 3:**
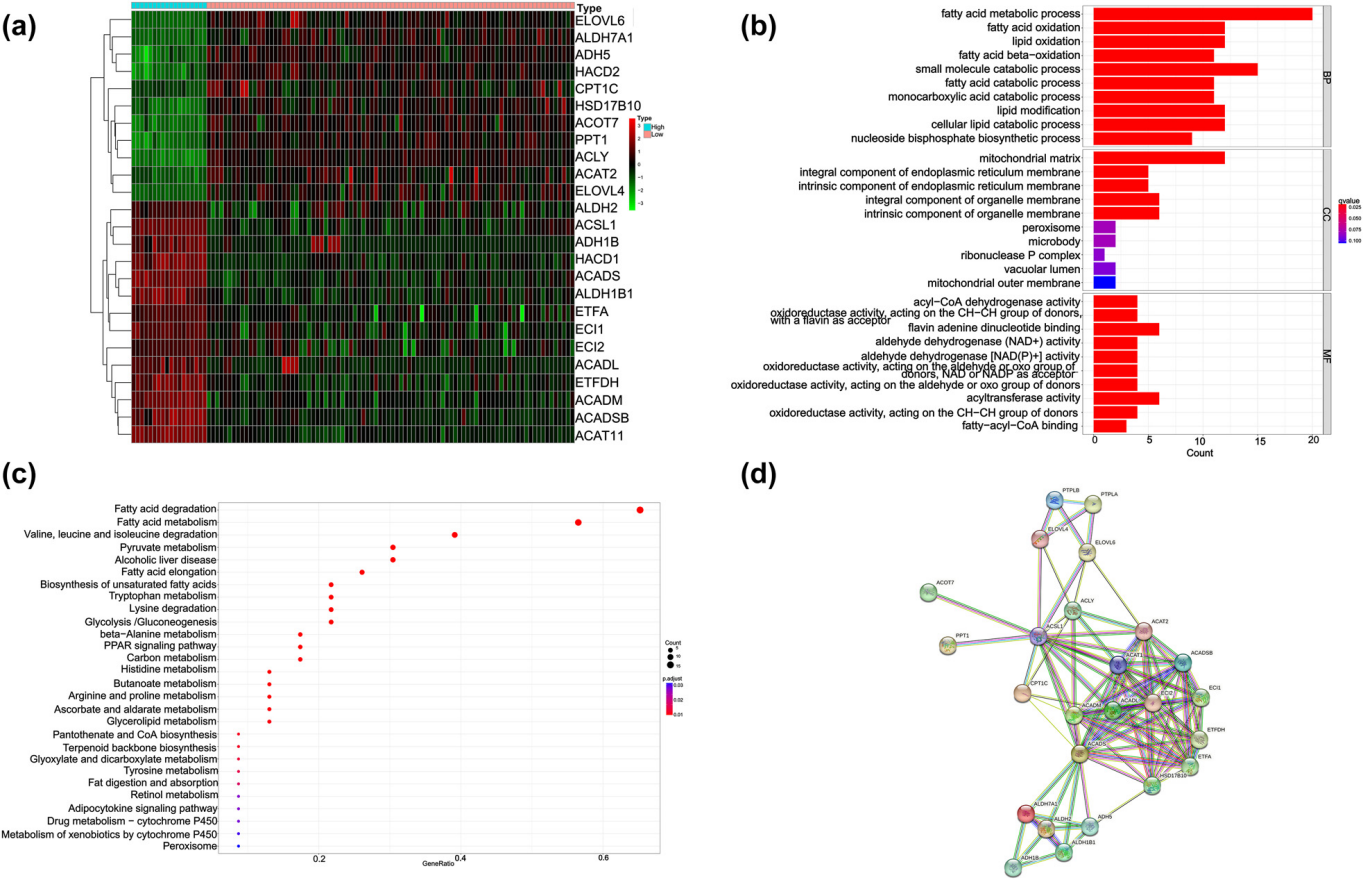
DEGs between the high- and low-risk groups. (a) Heatmap of DEGs between the high- and low-risk groups. (b) GO enrichment analysis in the high- and low-risk groups. (c) KEGG enrichment analysis in the high- and low-risk groups. (d) PPI network of the differential genes between the high- and low-risk groups.

It has a good prognosis in the training and testing databases. A total of 38 DEGs were identified between the two groups (Figure 3a), including 18 upregulated and 20 downregulated genes. Gene Ontology (GO) enrichment analysis revealed that these genes were involved in processes such as fatty acid metabolism, small molecule catabolism, fatty acid oxidation, lipid oxidation, lipid modification, cellular lipid catabolism, and the mitochondrial matrix (Figure 3b). KEGG enrichment analysis indicated that the DEGs were significantly enriched in pathways related to fatty acid degradation and the degradation of valine, leucine, and isoleucine (Figure 3c). To further understand the linkage of these 38 DEGs, we constructed a PPI network. In this network, 25 proteins encoded by differential genes interacted, including 95 edges (Figure 3d). Among the seven prognostic models that we constructed, ACADM, ADH5, ACSL1, ELOVL4, and ECI1 play a key role in the PPI network.

### 3.3 Different immune infiltration and immune checkpoints in the two groups

**Figure 4:**
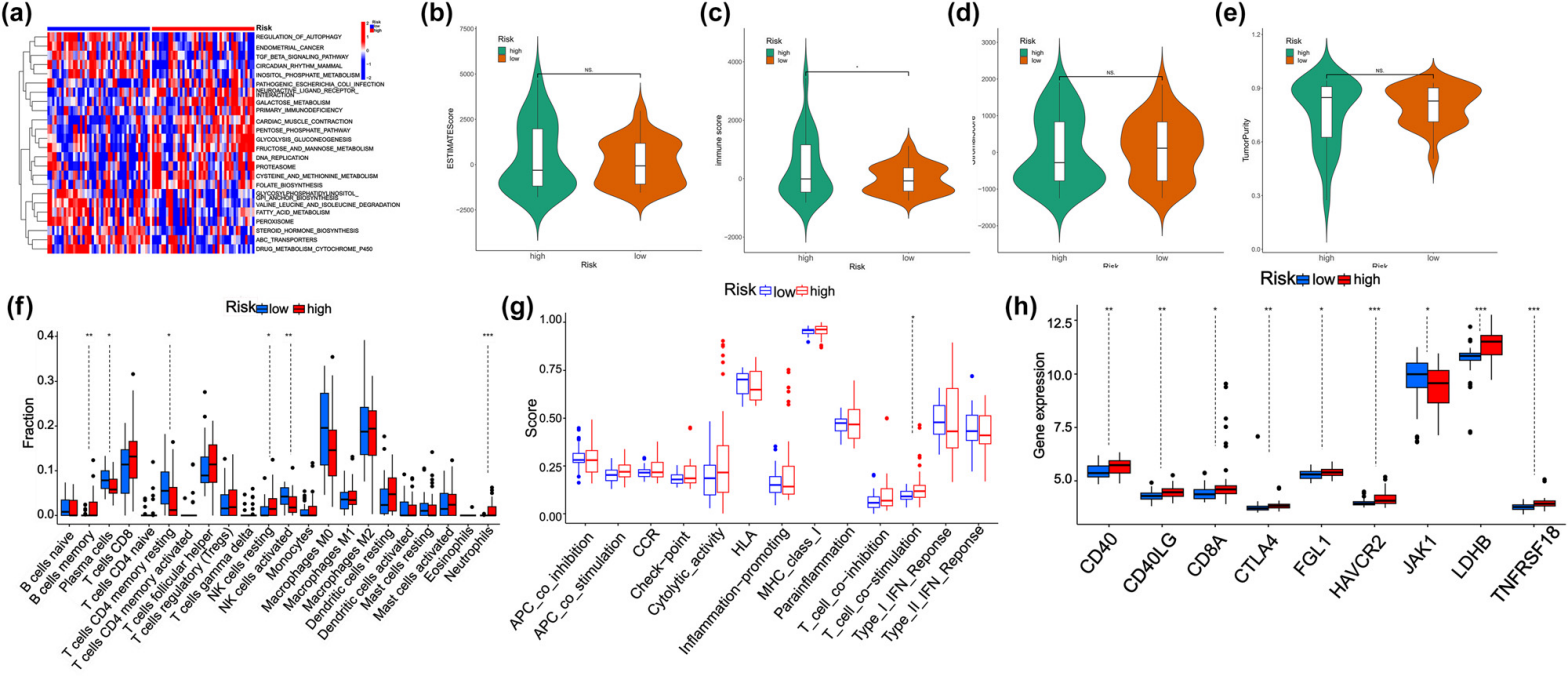
Analysis of immune infiltration and immune checkpoints in the high- and low-risk groups. (a) GSVA of each KEGG pathway in the high- and low-risk groups. (b)–(e) EWSTIMATE score, immune score, stromal score, and tumor purity of the high- and low-risk groups. (f) Immune cell infiltration analysis of the high- and low-risk groups. (g) Functional analysis of the high- and low-risk groups. (h) Immune checkpoint expression analysis of the high- and low-risk groups.

The high- and low-risk groups exhibit differences in the immune microenvironment. First, using the GSVA algorithm (Figure 4a), we found that there were significant differences in the enrichment of fatty acid metabolism pathways. The results indicated no significant difference in the stromal score, EWSTIMATE score, and tumor purity, except for the immune score of EWS patients (Figure 4b–e). CIBERSORT analysis revealed differences in the memory B cells, plasma cells, resting memory CD4 T cells, resting NK cells, activated NK cells, and neutrophils (Figure 4f). The functional analysis showed that there was a difference in T-cell costimulation-related functions (Figure 4g). Subsequently, the expression of immune checkpoints was analyzed, and it was found that there were differences in CD40, CD40LG, CD8A, CTLA4, FGL1, HAVCR2, JAK1, LDHB, and TNFRSF18, suggesting immunotherapy binding sites (Figure 4h).

### 3.4 Expression levels of PPT1 and ACOT7 are associated with the survival of EWS patients

**Figure 5:**
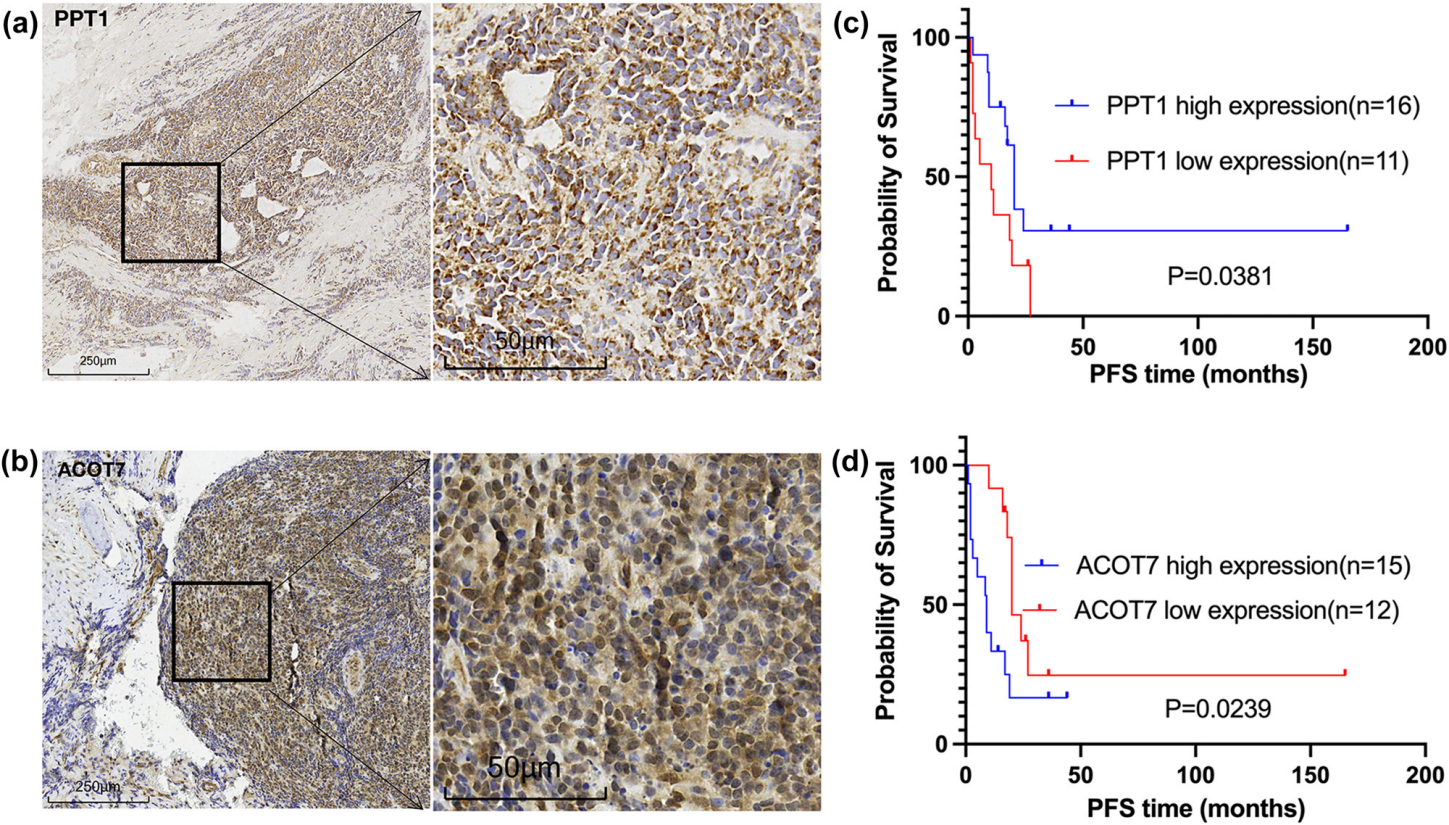
Immunohistochemical validation and statistical analysis of PPT1 and ACOT7. (a) PPT1 expression at magnifications of ×100 and ×400. (b) ACOT7 expression at magnifications of ×100 and ×400. (c) High expression of PPT1 was associated with a longer PFS time in patients with EWS (*P* = 0.0381). (d) Low expression of ACOT7 was associated with a longer PFS time in patients with EWS (*P* = 0.0239).

Combining the experiment and clinical information, the correlation between key markers and prognosis was verified. As shown in Figure 5a and b, the ACOT7 and PPT1 proteins were widely expressed in EWS tumor cells with strong cytoplasmic and nuclear staining; the remaining proteins (ACADM, ACSL1, and ECI1) are shown in Figure S2. To evaluate the relationship between ACOT7 and PPT1 expression levels and clinicopathological features, EWS specimens were divided into high-expression groups (*n* = 15 for ACOT7, *n* = 16 for PPT1) and low-expression groups (*n* = 12 for ACOT7, *n* = 11 for PPT1). The high ACOT7 expression levels were significantly associated with shorter PFS time (*P* = 0.0239; Figure 5d). The patients with low PPT1 expression levels were significantly associated with a shorter PFS time (*P* = 0.0381; Figure 5c). Cox regression analysis identified ACOT7 (*P* = 0.004) and PPT1 (*P* = 0.020) expression as independent prognostic indicators for PFS of EWS patients (Table 2). Subsequently, we validated the key markers in the GEO dataset GSE17674 (Figure S1).

**Table 2:** Multivariate Cox regression analysis based on the patient’s basic information

Variable	Multivariate Cox analysis		
	HR	95%CI	*P*
**Sex**			
Male	0.996	0.395–2.509	0.993
Female			
**Age**			
<18	0.703	0.236–2.096	0.527
≥18			
**Enneking stage**			
IIB, IIC	4.318	1.256–14.846	0.020
III			
**Location**			
Limbs	1.103	0.294–4.139	0.885
Trunk	0.578	0.181–1.850	0.356
Pelvis			
**PPT1 expression**			
High	0.284	0.098–0.823	0.020
Low			
**ACOT7 expression**			
High	5.796	1.778–18.891	0.004
Low			

## 4 Discussion

The treatment of EWS has not progressed for a long time. Recently, FAM has become an emerging target in cancer treatment. The growth of tumor tissue requires a large amount of energy consumption. By regulating FAM, tumor tissue can change its metabolism to meet this energy demand. Therefore, we hope to find the key target for regulating FAM in EWS and inhibiting tumor progression. We conducted bioinformatics analysis based on public data of EWS and FAMGs and screened seven DE-FAMGs in EWS with prognostic values. By collecting postoperative pathological sections from patients in our hospital and following up on patients, and performing statistical analysis of relevant data, immunohistochemical verification of the tumor tissues of patients and survival analysis, we selected two biomarkers related to the clinical prognosis of tumors, filling the gap in early treatment and prognosis judgement for EWS.

We found that ACOT7 and PPT1 are closely related to patient PFS by immunohistochemistry and correlation statistical analysis of EWS tissue. At present, many studies have shown that the genes we mentioned above participate in the pathogenesis of tumors by affecting lipid metabolism, but there is no relevant research in EWS. The disorder of fatty acid metabolism is considered a part of the malignant transformation in many different cancers [10,11]. Acyl coenzyme A thioesters (ACOT7) are the main subtype of the acyl coenzyme family, which can catalyse the hydrolysis of acyl-CoA to free fatty acids and coenzyme A without esterification. ACOT7 plays an important role in lipid metabolism [12,13,14]. ACOT7 plays a role in immune cell infiltration and is closely associated with the cancer immune microenvironment [15]. Some studies [14] have shown that ACOT7 can enhance the proliferation and migration of hepatocytes by increasing the content of C18:1 monounsaturated fatty acids. A study on lung cancer suggested that ACOT7 metabolites may influence cancer prognosis by mediating changes in fatty acid metabolism through endoplasmic reticulum stress [12]. Microarray analysis related to malignant melanoma also reported that tumor progression was related to the high expression of ACOT7 [16]. Methylated ACOT7 may play a role in tumor inhibition [17]. Overexpression of ACOT7 promotes the production of the monounsaturated fatty acid oleic acid, which enhances the proliferation and migration of liver cancer cells. [14]. Reports show that ACOT7 can inhibit lipid peroxidation by inhibiting ferroptosis [18]. Lysosomes play a dual role in cancer metabolism by facilitating catabolic processes such as autophagy and macropinocytosis while also promoting mTORC1-dependent anabolism. Palmitoyl protein thioesterase 1 (PPT1), a lysosomal enzyme, is overexpressed in various tumors and is associated with poor survival. However, this finding is inconsistent with our results (Figure 5c). This may be related to the fact that targeting PPT1 can block mTOR signal transduction while inhibiting autophagy and can be used as a target treatment [19]. Currently, the mechanism of action of ACOT7 and PPT1 in EWS has not been reported. We found for the first time that ACOT7 and PPT1 might be prognostic biomarkers in patients with EWS, and they are closely related to survival prognosis. Further experimental research and clinical validation are needed to understand how these factors influence fatty acid metabolism during the development and progression of EWS and their impact on patient prognosis.

The enrichment analysis results are consistent with the report of Buchou et al., indicating that FAMGs may be related to the modification of EWSR1 [20]. Immune infiltration and immune checkpoint analysis of the two groups were performed. Our analysis revealed significant differences in the memory B cells, activated NK cells, and neutrophils between the two groups. Research suggests that obesity can drive lipid accumulation in NK cells through the peroxisome proliferator-activated receptor (PPAR), resulting in the metabolic and functional “paralysis” of these cells. This impairs their ability to mount an effective antitumor response [21]. There have also been reports of NK cell-related fatty acid metabolism abnormalities leading to tumor progression in lung adenocarcinoma and lymphoma [22,23]. This may be related to the change in the tumor microenvironment (TME), leading to immune resistance [24]. Research has reported that when glucose utilization is restricted, tumor-induced oxidative neutrophils can maintain ROS production and inhibit T cells, leading to tumor development [25]. HAVCR2, LDHB, and TNFRSF18 have been a research basis for immune checkpoints. Studies have shown that an increase in glycolysis leads to the accumulation of lactic acid in cells, forming an acidic environment. This inhibits the activity of CD4 T cells and promotes the development of tumors. Therefore, overexpression of LDHB may represent a promising strategy to enhance the effectiveness of adoptive T-cell transfer therapy [26]. The genes HAVCR2 and TIM-3 (t cell immunoglobulin domain and mucin domain 3, CD366) have been shown to be coexpressed with PD-1. Their high expression may block the adaptive resistance of PD-1 to promote tumor progression, while blocking the TIM-3 and PD-1 pathways significantly inhibits tumor growth [27]. Some studies have shown that TNFRSF18 (glucocorticoid-induced tumor necrosis factor) can activate the MAPK/ERK and NF-kB pathways through the high-level expression of T-reg cells, thus leading to the production of proinflammatory cytokines and enhancing the antitumor effect [28]. However, the specific immune mechanisms underlying EWS remain unclear, highlighting the need for further investigation.

In this study, database analyses were conducted to identify FAMG targets that may influence the prognosis of EWS. However, due to the low prevalence of EWS, the database contains a small number of datasets and lacks a comprehensive multicenter, multidata set study. Further experimental validation is also lacking, and further research is needed. In subsequent research, it is necessary to increase the sample size and further study the mechanism. In addition, an increasing number of studies are exploring levels of tumors using Multiomics. We can also conduct research on EWS from other aspects such as single-cell and proteomics to understand the specific mechanisms of fatty acid metabolism in disease occurrence, development, and drug resistance.

## 5 Conclusions

In summary, we developed a prognostic model for EWS and identified two biological targets based on public databases and patient data from our hospital. Our study identifies new biological targets for predicting the prognosis of EWS patients, potentially aiding in clinical decision-making.

## Abbreviations

EWS: Ewing’s sarcoma
GEO: Gene Expression Omnibus
ICGC: International Cancer Genome Consortium
ROS: Reactive oxygen species
FAMGs: Fatty acid metabolism-related genes
GO: Gene Ontology
KEGG: Kyoto Encyclopedia of Genes and Genomes
PPI: Protein–Protein interaction
GSVA: Gene set variation analysis
PPAR: Peroxisome proliferator-activated receptor
TME: Tumor microenvironment

## Supplementary Material

Supplementary material
