# Supplementary material for: A prognostic model correlated with fatty acid metabolism in Ewing’s sarcoma based on bioinformatics analysis
Source: Open Med (Wars). 2025 Aug 7;20(1):20251238. doi: 10.1515/med-2025-1238 (PMC12355353; doi:10.1515/med-2025-1238)
Supplement: Supplementary material [file med-2025-1238-sm.pdf]

Supplementary material

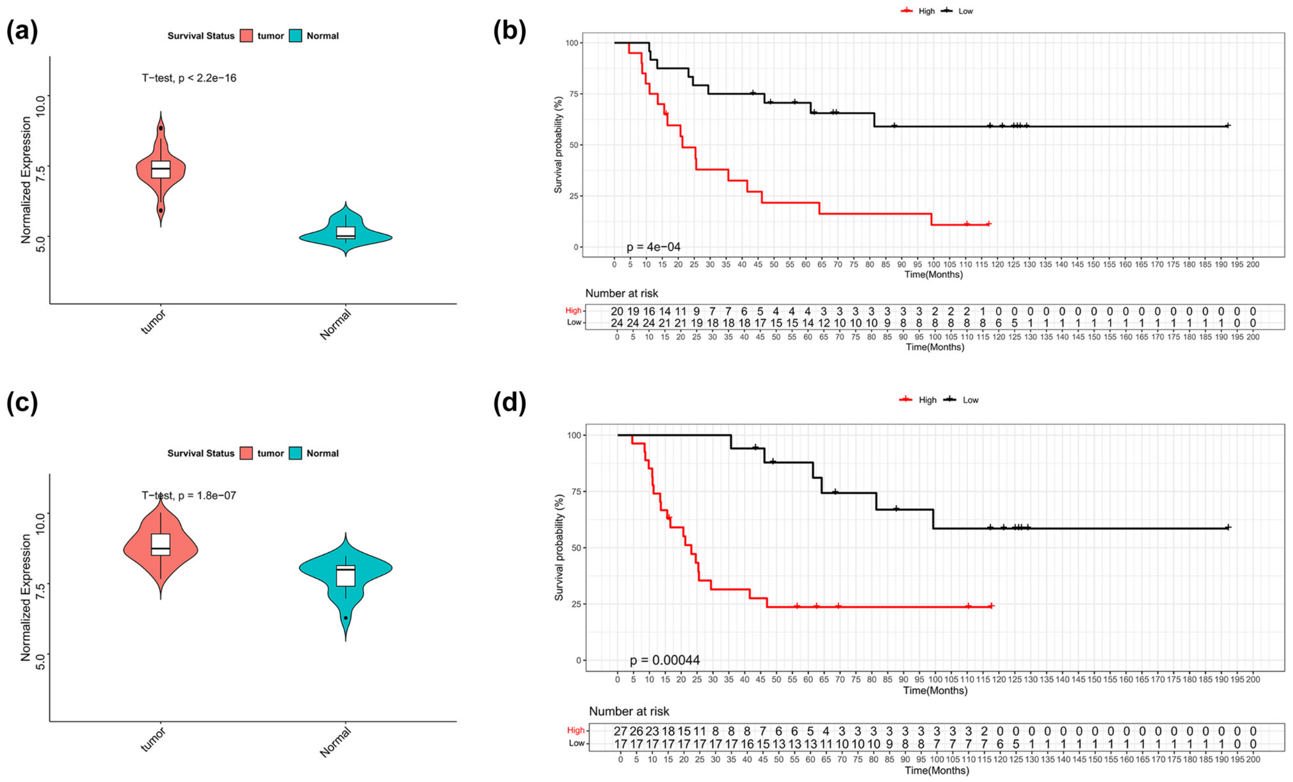

Figure S1: The key markers validated in the GEO dataset. (a) Expression of ACOT7 between Tumor and Normal in GSE17674. (b) Survival curves of ACOT7 in high and low expression groups. (c) Expression of PPT1 between Tumor and Normal in GSE17674. (d) Survival curves of PPT1 in high and low expression groups.

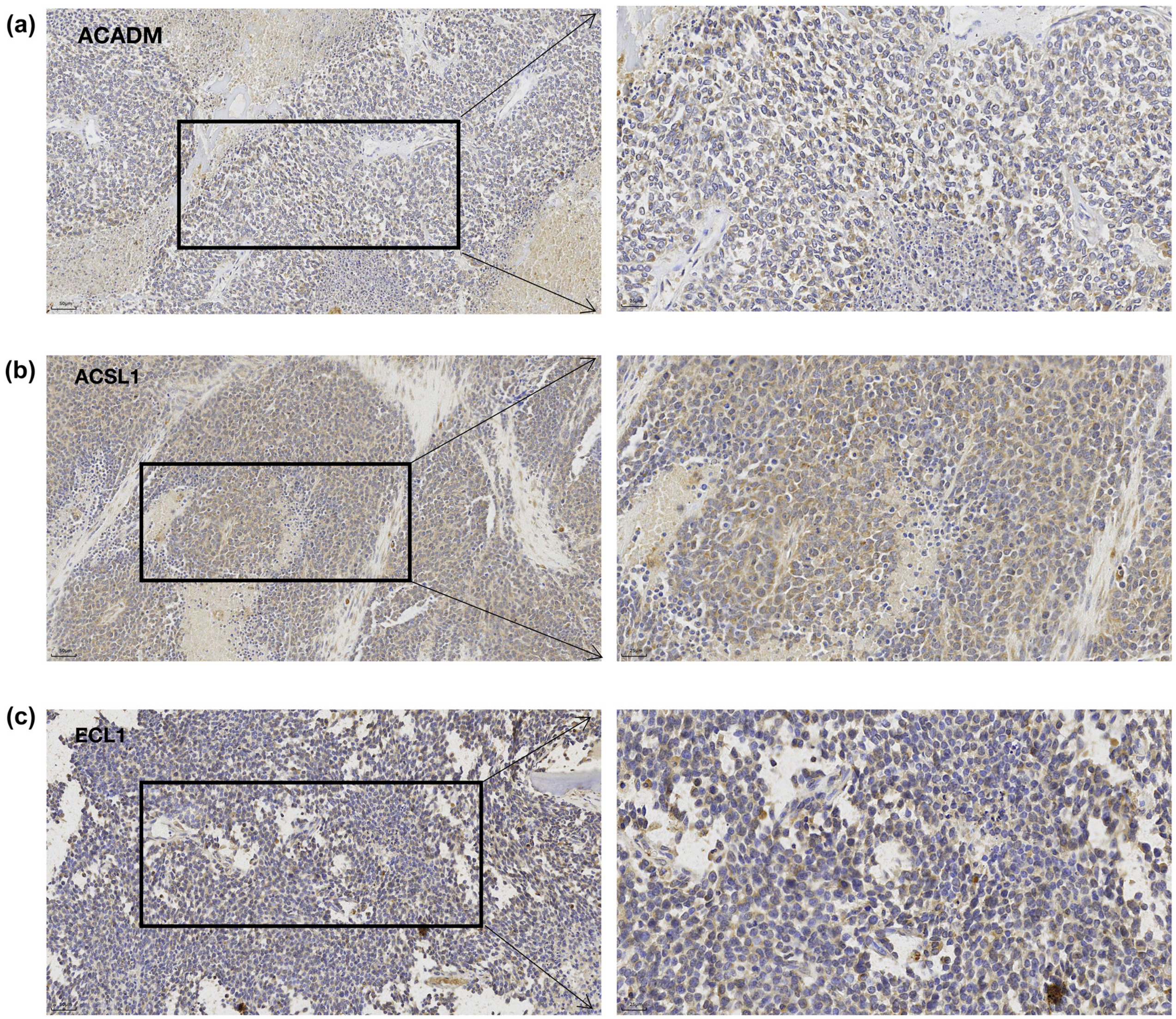

Figure S2: Immunohistochemical of ACADM, ACSL1 and ECI1. (a) ACADM Expression of tumor. (b) ACSL1 Expression of tumor. (c) ECI1 Expression of tumor.

Table S1: Antibody information of 5 protein encoded by related genes in the prognostic model

Antibody	Supplier	Product No.	Species	Dilution ratio	Dilution
ACOT7	Proteintech	15972-1-APProteintech	Human	1:200	2%BSA
ACSL1	Affinity	DF1389-1-AP	Human	1:150	2%BSA
ECL1	Affinity	DF6343	Human	1:150	2%BSA
PPTI	Affinity	DF7733	Human	1:150	2%BSA
ACADM	Affinity	DF6670	Human	1:50	2%BSA

Table S2: DE-FAMGs in EWS. 25 differentially expressed genes related to fatty acid metabolism between EWS and normal tissues were selected from GEO's public database for further analysis. Among them, there are 11 up-regulated and 14 down-regulated genes

id	logFC	AveExpr	t	P.Value	adj.P.Val	B
ACAT1	−1.241016	5.03429535	−23.666506	2.90×10^{-44}	2.41×10^{-42}	90.3062216
ACLY	2.1364052	7.10431102	21.6559829	7.49×10^{-41}	3.11×10^{-39}	82.4299751
HACD1	−1.7820429	4.5088635	−16.83025	8.41×10^{-32}	2.33×10^{-30}	61.5285363
ACADSB	−1.6162235	4.38715477	−16.515282	3.63×10^{-31}	7.52×10^{-30}	60.0624688
ACADS	−1.3425117	5.66886158	−15.251596	1.45×10^{-28}	2.40×10^{-27}	54.0570392
PPT1	2.11337585	8.76849903	14.5716411	3.93×10^{-27}	5.44×10^{-26}	50.7466176
ACADM	−1.5949001	8.35930074	−14.353525	1.15×10^{-26}	1.19×10^{-25}	49.673529
HACD2	1.61336889	5.76227523	14.08609	4.30×10^{-26}	3.96×10^{-25}	48.3506645
ALDH1B1	−1.1020159	4.41120846	−13.712227	2.75×10^{-25}	2.29×10^{-24}	46.4885995
ETFDH	−1.7788073	5.56772005	−13.599817	4.83×10^{-25}	3.64×10^{-24}	45.9259126
ADH5	1.26338124	7.94595491	13.3032624	2.14×10^{-24}	1.48×10^{-23}	44.4354749
ACOT7	1.39076576	6.63365077	12.9959669	1.01×10^{-23}	6.44×10^{-23}	42.8822634
ACSL1	−3.0509821	6.25926207	−12.261093	4.26×10^{-22}	2.52×10^{-21}	39.1350065
HSD17B10	1.36984333	8.42326373	12.1137517	9.06×10^{-22}	5.01×10^{-21}	38.3786939
ELOVL6	1.50187678	4.24214831	10.510398	3.66×10^{-18}	1.79×10^{-17}	30.0750299
ALDH7A1	2.58734132	6.90968782	10.2791001	1.22×10^{-17}	5.63×10^{-17}	28.8711739
ELOVL4	2.06084472	4.74089609	9.56160214	5.14×10^{-16}	2.13×10^{-15}	25.1402326
ADH1B	−1.6861777	3.59191842	−8.9747169	1.08×10^{-14}	4.27×10^{-14}	22.1034282
ECI1	−1.1726424	6.69758624	−8.6925066	4.64×10^{-14}	1.68×10^{-13}	20.652072
ACADL	−1.2342565	3.70619382	−8.1992954	5.83×10^{-13}	1.86×10^{-12}	18.135805
ACAT2	1.23663676	6.52647445	7.77061877	5.13×10^{-12}	1.52×10^{-11}	15.9761029
ETFA	−1.2415298	8.01043732	−6.2854997	7.28×10^{-9}	1.59×10^{-8}	8.79752013
ECI2	−1.431416	6.98837336	−5.6053118	1.64×10^{-7}	3.25×10^{-7}	5.73628124
CPT1C	1.48043662	5.24010346	5.5598326	2.01×10^{-7}	3.88×10^{-7}	5.5381457
ALDH2	−1.213117	7.60927159	−4.7570839	6.19×10^{-6}	1.05×10^{-5}	2.19899244
